# Divergent and convergent roles for insulin-like peptides in the worm, fly and mammalian nervous systems

**DOI:** 10.1007/s10158-013-0166-9

**Published:** 2014-01-07

**Authors:** Hiu E. Lau, Sreekanth H. Chalasani

**Affiliations:** 1 Division of Biological Sciences, University of California San Diego, La Jolla, CA 92093 USA; 2 Molecular Neurobiology Laboratory, The Salk Institute for Biological Studies, La Jolla, CA 92037 USA

**Keywords:** Insulin, *C. elegans*, *D. melanogaster*, Mammals, Nervous system, Behavior

## Abstract

Insulin signaling plays a critical role in coupling external changes to animal physiology and behavior. Despite remarkable conservation in the insulin signaling pathway components across species, divergence in the mechanism and function of the signal is evident. Focusing on recent findings from *C. elegans*, *D. melanogaster* and mammals, we discuss the role of insulin signaling in regulating adult neuronal function and behavior. In particular, we describe the transcription-dependent and transcription-independent aspects of insulin signaling across these three species. Interestingly, we find evidence of diverse mechanisms underlying complex networks of peptide action in modulating nervous system function.

## Background

Insulins are small peptide hormones with diverse and wide-ranging roles in regulating multiple aspects of animal physiology. Originally purified from ground and filtered dog pancreas, the significance of insulin became quickly apparent when Frederick Banting and his laboratory assistant, Charles Best, used this extract to treat children suffering from diabetic ketoacidosis (Bliss 1993). This pioneering work led to the 1923 Nobel Prize in Medicine and Physiology being awarded to Banting and his mentor John Macleod. Fifty years passed before the insulin receptor was identified (Freychet et al. 1971; Kahn et al. 1974). Since these pioneering studies, researchers have begun to unravel the role of insulin signaling in regulating metabolism, particularly acting as an anorexigenic signal that suppresses appetite (Woods et al. 1996). Specifically, mammalian insulin released from pancreatic beta cells during satiated states acts in the brain to reduce food intake and activate catabolic pathways reducing weight gain (Woods et al. 1996). In the brain, insulin acts on a complex network of neurons in the hypothalamic arcuate nucleus and affects feeding states (reviewed in Fernandez and Torres-Aleman 2012). Beyond its classical role in regulating glucose levels, insulin also regulates longevity, animal development and neuronal functions (Kimura et al. 1997). In the context of development, peptides of the insulin superfamily regulate neuronal proliferation, survival and neurite outgrowth (Hodge et al. 2004; Barres et al. 1992; Torres-Aleman et al. 1994; Ozdinler and Macklis 2006). Moreover, insulin signaling modulates neural circuits and influences their output, as measured by behavior and neuronal activity in a number of model organisms (Chen et al. 2013; Oda et al. 2011; Chalasani et al. 2010; Root et al. 2011; Marks et al. 2009; Ahmadian et al. 2004; Leinwand and Chalasani 2013).

**Fig. 1:**
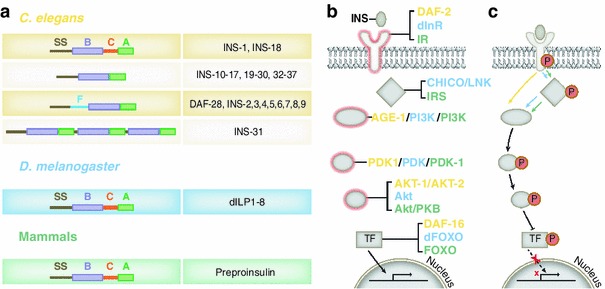
**a** Structure of *C. elegans*, *D. melanogaster* and mammalian insulin/ILPs. There have been 40 ILP genes identified in *C. elegans* (INS-1 to INS-39 and DAF-28). INS-1 is most similar to mammalian insulin. In worms, ILPs are generated as various combinations of signal sequence (*SS*), A-chain (*A*), B-chain (*B*), and C-peptide (*C*). A worm-specific F-peptide (*F*) is also found in a subset of *C. elegans* ILPs. *D. melanogaster* dILPS and mammalian preproinsulin peptide have a similar structure, containing a signal sequence, B-chain, C-peptide and A-chain. **b** Basal state of the insulin signaling pathway. Mammalian insulin signaling components are labeled in *green*. *C. elegans* and *D. melanogaster* homologs of these molecules are shown in *yellow* and *blue,* respectively. Insulin receptors are conserved receptor tyrosine kinases. Upon ligand binding, insulin receptors phosphorylate their substrate, IRS, which in turn activates PI3K. In contrast to flies and mammals, the worm IRS (called IST-1) seems to work in parallel to the AGE-1/PI3K pathway shown here. Two kinases PDK-1 and AKT act downstream of PI3K and phosphorylate the forkhead transcription factor, FOXO, preventing it from activating target genes. In the absence of insulin, FOXO enters the nucleus and promotes the transcription of target genes. **c** Activation of insulin signaling pathway inhibits transcription of target genes. Activation of the insulin receptor results in phosphorylation of the insulin receptor substrates (CHICO and LNK in *D. melanogaster*). Phosphorylation of IRS activates downstream kinases PI3K, PDK and AKT, leading to the inhibition of FOXO, thereby reducing the transcription of target genes

The molecular components of the insulin signaling pathway have been well characterized in worms, flies and mammals (Fig. 1). Insulin or insulin-like peptides (ILPs) bind and activate a tyrosine kinase like insulin receptor (Massague et al. 1980). Upon activation, the insulin receptor phosphorylates a group of insulin receptor substrate (IRS) proteins, which in turn activate phosphoinositide 3-kinase (PI3K). When activated, PI3K regulates the activity of downstream kinases Akt/protein kinase B (PKB), leading to phosphorylation of the forkhead transcription factor, FOXO. Once phosphorylated, FOXO is unable to enter the nucleus, thereby reducing the transcription of target genes (Accili and Arden 2004). In this review, we focus on insulin signaling pathways involving FOXO-dependent transcription. However, we also note that in mammals, insulin signaling can activate additional downstream pathways involving MAP kinase and the ETS family of transcription factors (Langlais et al. 2004). In summary, one way in which insulin signaling regulates animal physiology is by inhibiting transcription of FOXO-dependent target genes.

The molecules involved in the canonical insulin signaling cascade are highly conserved across both vertebrates and invertebrates. The *C. elegans* homologs of the insulin receptor, PI3K and FOXO are called DAF-2, AGE-1 and DAF-16, respectively (Fig. 1) (Pierce et al. 2001; Paradis et al. 1999; Morris et al. 1996; Lin et al. 1997; Paradis and Ruvkun 1998). However, the *C. elegans* homolog of IRS (IST-1) is proposed to function in a parallel pathway to the AGE-1/PI3K pathway (Wolkow et al. 2002). The other well-studied invertebrate model, *D. melanogaster*, has all components of the mammalian insulin signaling pathway including an insulin receptor (dInR), IRS (CHICO and LNK) and FOXO (dFOXO) with similar functions (Staveley et al. 1998; Leevers et al. 1996; Poltilove et al. 2000; Werz et al. 2009). The homologs of insulin signaling molecules found across both vertebrates and invertebrates exemplify the remarkable conservation of this important pathway through evolution.

In this review, we will discuss the role of insulin signaling in regulating adult neuronal function. We highlight the similarities and differences between the mechanisms employed by both vertebrates and invertebrates to modulate neuronal activity. Interestingly, although there is large variability in the number of insulin ligands across species, there is remarkable conservation of the components used in the insulin signaling cascade. For the scope of this review, we limit our discussion to the members of the insulin/ILP gene superfamilies, including 40 *C. elegans* ILP genes, 8 dILP genes, and mammalian insulin and insulin-like growth factor (IGF) genes. We describe divergence in the roles of insulin and summarize the mechanisms by which insulin signals are regulated to achieve specificity in function.

## Insulin signaling in the nematode, *C. elegans*

**Fig. 2:**
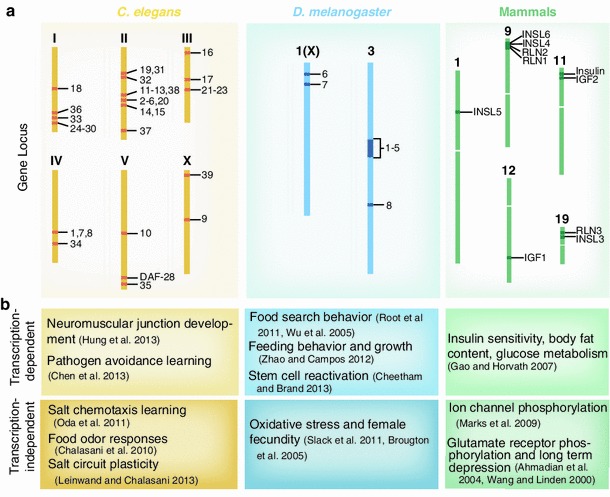
**a** Chromosomal location of insulin/insulin-like peptide genes in *C. elegans*, *D. melanogaster* and humans. *C. elegans* genome has 40 ILP genes spread across all six chromosomes. *D. melanogaster* has eight DILP genes that are found on chromosomes 1(X) and 3. The human insulin superfamily has ten members including insulin, IGF1 and IGF2, relaxins 1-3 (RLN1-3), and insulin-like peptides 3-6 (INSL3-INSL6). **b** Insulin signaling acts through transcription-dependent and transcription-independent mechanisms to regulate metabolism, development and neuronal function

The well-studied nematode, *C. elegans,* has provided critical insights into insulin signaling and its role in regulating animal physiology, longevity and neuronal functions. About 40 ILP genes (*ins*-*1* to *ins*-*39* and *daf*-*28*) have been identified in the genome (Ritter et al. 2013). Characteristic of gene duplication events throughout evolution, these ILP genes are distributed across all six pairs of chromosomes in the worm as shown in Fig. 2a. The expansive insulin gene family allows for both divergence and redundancy in the function of this crucial signaling network. INS-1 is most similar to the mammalian insulin peptide, but the other worm ILPs also show conserved structural domains with human insulin A- and B-chains (Fig. 1a) (Pierce et al. 2001). As described above (Fig. 1b), ILPs bind the conserved tyrosine kinase receptor DAF-2 (Kimura et al. 1997) and act via AGE-1 and AKT kinases to phosphorylate DAF-16 and activate the transcription of target genes (Paradis et al. 1999; Morris et al. 1996; Lin et al. 1997; Paradis and Ruvkun 1998). The large number of ILPs emphasizes the diversity in signaling roles for these ligands in regulating physiology.

Recent studies analyzing the role of ILPs in the *C. elegans* nervous system have identified two flavors of this signaling pathway: a transcription-*dependent* and a transcription-*independent* component. Recent work shows that insulin signaling requires a transcription-dependent process in regulating the development of the neuromuscular junction (Hung et al. 2013). In this context, multiple insulins (INS-6 from ASI sensory neurons and INS-4 from both ASI sensory and motor neurons) act on the DAF-2 receptor to regulate the effects of a transcription factor, F-box factor (FSN-1), on neuromuscular junction morphology and motor neuron–muscle synapse numbers. *fsn*-*1* mutants have aberrant synapse numbers and morphology, and these effects are rescued by specifically reducing insulin signaling in the post-synaptic muscle. These results show that insulin signals likely antagonize FSN-1 signaling to regulate neuromuscular synapses. In this example, insulin signaling interacts with another neuronal signaling pathway to fine tune a key developmental step using a transcription-dependent component.

Interestingly, INS-6 released from the ASI neurons can also function outside of the neuromuscular junction, for responding to pathogenic bacteria. *C. elegans* exposed to pathogenic bacteria can learn and avoid that pathogen upon a second exposure (Zhang et al. 2005). In the pathogen-avoidance learning paradigm, ASI sensory neuron-released INS-6 peptide inhibits the transcription of INS-7 peptide in the oxygen-sensing URX neurons. In the absence of INS-6, INS-7 acts through the DAF-2 receptors to regulate the localization of DAF-16 in downstream RIA interneurons (Chen et al. 2013). This ILP–ILP loop provides another example of the complexity and diversity of signaling used to achieve specific changes in neural networks. Moreover, these results also show that the same ligand, INS-6 released from the same ASI neuron, can have multiple distinct roles in the nervous system. Additionally, INS-6 has also been shown to play a crucial role in coupling environmental conditions with development by inhibiting dauer entry and promoting dauer exit (Cornils et al. 2011). In response to harsh environmental conditions, *C. elegans* larvae enter a reversible stage of growth arrest (termed “dauer”) wherein the animal arrests feeding and limits locomotion (Cassada and Russell 1975). For regulating dauer arrest, the INS-6 signal is also released from ASI sensory neurons. However, an extra pair of sensory neurons, the ASJs, is also involved in INS-6 production in the context of dauer arrest (Cornils et al. 2011). Collectively, these results suggests that a high degree of spatial and temporal control of insulin release enables the same ligand to perform different roles based on context.

Results from a number of laboratories have shown that insulins can also modulate neuronal activity in a transcription-independent manner that occurs on faster timescales. In a salt chemotaxis learning paradigm, a 10–60-min exposure to a particular concentration of salt in the absence of food leads to reduced attraction to that salt concentration (Tomioka et al. 2006). INS-1, DAF-2 and AGE-1 all play roles in altering neural activity of the salt-sensing ASE neurons (Oda et al. 2011). Importantly, this process does not require DAF-16 or transcription. Similarly, INS-1 released from AIA interneurons has been shown to suppress AWC sensory neuron responses to food odors (Chalasani et al. 2010). In both of these examples, INS-1 acts on short timescales: less than a second in the case of AWC responses and a few minutes in ASE-associated behavior. These results show that insulin signaling can also function independently of transcription in influencing neuronal function for fast neural modulation.

Recent work from our laboratory demonstrated that the source of the insulin ligand is also crucial in determining its function. We showed that in contrast to ASI-released INS-6, ASE neurons use a proprotein convertase BLI-4 to process INS-6 and recruit AWC sensory neurons into the salt neural circuit (Leinwand and Chalasani 2013). In this example, INS-6 signaling functions on a short timescale of less than one second and likely do not require transcription. These results argue that the same ligand (INS-6) can be processed by different machinery in different neurons and function in a transcription-dependent and -independent manner based on context. In summary, modulation of neural function by insulin signaling is regulated at many levels: at the source and target, spatially and temporally and through transcription-dependent and -independent mechanisms.

## Insulin signaling in the fruit fly, *D. melanogaster*

Similar mechanisms and functions of insulin peptides have been observed in another invertebrate model organism, *D. melanogaster*. Genome analysis has revealed eight putative *Drosophila* insulin-like peptide (dILP) precursors with structure similar to mammalian insulin (Brogiolo et al. 2001; Colombani et al. 2012). These eight genes are found on two chromosomes as shown in Fig. 2a. *dILPs 1*-*5* are found on the third chromosome as a gene cluster, *dILP*-*8* is on the third chromosome at a separate locus while *dILP 6* and *7* are on the X-chromosome as separate loci (Brogiolo et al. 2001; Colombani et al. 2012). Orthologs to the mammalian and *C. elegans* insulin pathway have also been identified including a *dInR* (*Drosophila* insulin receptor), *chico* and *lnk* (IRS), PI3K and *akt* (Protein kinase B) (Staveley et al. 1998; Leevers et al. 1996; Poltilove et al. 2000; Werz et al. 2009). Complex expression of dILP*s* in *D. melanogaster* is spatially and temporally regulated. In relation to modulating neural function and behaviors, the median neurosecretory cells in the fly brain express a subset of dILPs (Brogiolo et al. 2001; Rulifson et al. 2002). Ablation of insulin producing cells results in growth defects and misregulated carbohydrate concentration in the hemolymph (Rulifson et al. 2002).

Similar to results obtained using the *C. elegans* model, insulin signaling in *D. melanogaster* has also been shown to regulate multiple aspects of neuronal development. During development, a small cluster of median neurosecretory cells release insulin into the circulatory system in response to nutrients (Ikeya et al. 2002). Circulating insulin acts on neurons in the mushroom body and regulates the feeding behavior and growth of the fly larvae (Zhao and Campos 2012). In this context, the source and identity of dILPs acting on the mushroom body remains unclear. dILPs also play an important role in the dynamic regulation of stem cell growth in response to metabolic changes. During development, quiescent neural stem cells in the brain must be reactivated for proliferation to generate adult neurons. Nutritional status is sensed by glial cells that express and secrete dILPs. Locally secreted dILP2 and dILP6 from glia reactivates neural stem cells by acting on the PI3K/Akt pathway (Cheetham and Brand 2013). In summary, these studies demonstrate the role of dILPs in coupling nutritional status to neural development and growth.

Insulins have also been shown to couple environmental conditions to physiology in the adult fly. *D. melanogaster* S6 kinase is involved in modulating hunger response by regulating the opposing effects of insulin and neuropeptide F signaling pathways (Wu et al. 2005). Low levels of circulating insulin leads to the increase in the expression levels of the short neuropeptide F receptor at the synapse between the olfactory receptor neuron and the projection neuron. This transcription-dependent event, which is inhibited by insulin signaling, enhances the attraction of adult flies to food (Root et al. 2011). These results show that dILPs produced in the brain couple nutritional status with neural circuit functions. In contrast to *C. elegans* findings of fast transcription-independent action of ILPs, the timescale of insulin action in flies can range from hours to days to accommodate changing nutrient availability. Not surprisingly, many of these dILP actions require dFOXO and transcription. To summarize, these studies show that regardless of location and timescale, insulin represents a versatile signal that works with other peptide signaling systems to integrate nutritional status to regulate neuronal development and function.

In some cases, dILP signaling outside of the nervous system does not involve dFOXO. Mutations in dFOXO do not abrogate the increase in oxidative stress resistance upon exposure to paraquat seen in animals with reduced insulin signaling (Slack et al. 2011; Broughton et al. 2005), suggesting a transcription-independent role for insulin. Moreover, dFOXO does not seem to be required to promote fecundity, which in turn is influenced by insulin signaling (Slack et al. 2011). Together, studies of longevity phenotypes provide support that insulin signaling uses diverse downstream mechanisms for regulating somatic changes. Molecular machinery other than dFOXO can also mediate somatic changes when insulin signaling is involved.

## Insulin signaling in mammals

Compared to the findings in invertebrates, mammalian insulin signaling relies on similar molecular pathway components but has striking differences in function and mechanism. The human insulin superfamily consists of ten members including a single insulin (Bell et al. 1980), IGF1 and IGF2 (Rinderknecht and Humbel 1978), relaxins (Bedarkar et al. 1977), relaxin-like growth factors (Bullesbach and Schwabe 1995) and an early placental insulin-like peptide (Chassin et al. 1995). We focus our discussion of neural modulation and behavior on the role of three genes: insulin, IGF1 and IGF2 (Bell et al. 1980; Harper et al. 1981; Owerbach et al. 1980). Similar to the rat and mouse insulin genes, the mammalian insulin gene encodes a preproinsulin peptide, which has an A-chain, B-chain, C-peptide and signal sequence (Murray-Rust et al. 1992). To become functionally active, the signal sequence and C-peptide are cleaved, leaving the A- and B-chains that interact with disulfide bonds to form functional insulin (Murray-Rust et al. 1992). Although IGF1 and IGF2 share similarity in structure to insulin (Rinderknecht and Humbel 1976), they bind distinct receptors. Insulin, IGF1 and IGF2 bind the insulin receptor, IGF1 receptor (IGF1R) and IGF2 receptor (IGF2R), respectively (Vashisth and Abrams 2010; Hawkes and Kar 2004).

Peripheral insulin made by the pancreas can cross the blood brain barrier and influences the nervous system (Banks 2004). Although the insulin transporter remains unknown, studies have shown that insulin transport across the blood brain barrier is regulated and saturable (Banks 2004). Similar to developmental roles of ILPs in invertebrate animals, sensory map formation in rodents also requires IGF1 and IGF1R. During rat development, IGFs act as chemoattractants to guide the projection of olfactory neurons in the olfactory bulb (Scolnick et al. 2008). Similar to the role of ILPs in invertebrate physiology, ILPs in mammals also play a role in regulating neuronal growth and development (Chiu and Cline 2010).

The mechanism by which insulin and IGFs modulate neuronal function differs from their roles in the pancreas. In the brain, insulin mediates transcription-independent changes in specific neuronal populations on shorter timescales when compared to other tissues. Insulin in the olfactory bulb suppresses the activity of the shaker-like voltage-gated potassium channel, Kv1.3. Intranasal delivery of insulin resulted in increased phosphorylation of the Kv1.3 channel, leading to improved memory in recognition tasks and increased odor discrimination (Marks et al. 2009). These results link insulin signaling in the olfactory bulb to higher-order brain functions. In a similar transcription-independent manner, insulin and IGF1 both induce long-term depression and attenuate AMPA-mediated currents in the cerebellum through endocytosis of receptor subunits (Ahmadian et al. 2004; Wang and Linden 2000). On the timescale of seconds, IGF1 also induces increases in calcium channel currents in a process that requires PI-3K (Blair and Marshall 1997). On these fast timescales, insulin in the rodent brain modulates neuronal function in a transcription-independent manner by modulating the activity of ion channels (Marks et al. 2009; Blair and Marshall 1997).

Transcription-dependent modulation of the nervous system through insulin signaling has also been observed in mammals. In one study, insulin signaling acts in the hypothalamus to regulate the activity of another forkhead transcription factor, FOXA2 (Silva et al. 2009). Insulin signaling inhibits activity of FOXA2, reducing expression of neuropeptide genes orexin and melanin-concentrating hormone. However, unlike in the invertebrate examples, this action of insulin leads to physiological effects outside the nervous system. Changes in orexin and melanin-concentrating hormone modify insulin sensitivity, body fat content and glucose metabolism of the animal. Although transcription-dependent action of insulin exists, these effects mainly affect metabolism and the activity of cell types outside the nervous system. In the mammalian system, it is likely that a complex network of many different hormones and signaling peptides takes the place of an elaborately evolved insulin system observed in worms.

## Conclusions

There is remarkable conservation in the components of the insulin signaling pathway across worms, flies and mammals. In addition to conserved molecular machinery, convergence in function of this signaling pathway is also evident. During neural development, insulin signals play an important role in synapse formation, neural stem cell regulation and neuronal growth (Zhao and Campos 2012; Cheetham and Brand 2013; Scolnick et al. 2008; Hung et al. 2013). We have highlighted both transcription-independent and transcription-dependent actions of insulin regulating neuronal function in invertebrate and vertebrate animals. A recurring theme in insulin function across species is the critical role that the signal plays in coupling different aspects of physiology to changing environmental conditions.

However, evolution of individual species over time has resulted in divergence in some of the mechanisms underlying ILP function. In particular, the number of peptides in the ILP superfamily varies across species. Branching of the insulin signaling pathway is also evident when we compare the broader network of peptide signaling pathways that interact with insulin signaling. In place of an elaborate insulin superfamily seen in invertebrates, mammals employ a complex network of signals that do not involve overlapping signaling components. To encode nutritional status, molecules such as neuropeptide Y, leptin, ghrelin, corticotropin-releasing hormone (CRH) and melanin-concentrating hormone are used to modify specific target neurons in the hypothalamus (Gao and Horvath 2007). The multitudes of signals allow the mammalian nervous systems to integrate nutritional status effectively. For example, leptin, like insulin, also signals satiety using a different signaling pathway to regulate body weight and feeding behavior (Baskin et al. 1999). Similar to transcription-dependent roles of insulins, leptin acts by stimulating CRH gene expression (Schwartz et al. 1996). There are some examples of a similar peptide–peptide interaction in invertebrates as well. Specifically, dILPs regulate neuropeptide Y-like signaling in *D. melanogaster* (Root et al. 2011; Wu et al. 2005). Although the repertoire of peptide signals and accompanying functions differ in invertebrates, a common theme of complex peptide networks can be observed both in invertebrate and vertebrate species.

Complexity in ILP signaling accompanies this diverse superfamily of molecules as combinations of ILPs can be used together in many different contexts. For example, with more than 40 ILPs in the worm (Ritter et al. 2013), combinations of peptides are used to regulate animal physiology (Chen et al. 2013). Moreover, interactions with non-ILP signals can also generate additional control over animal physiology. One intriguing question remains to be answered: How are these ILPs regulated to carry out specific functions? Controlling the source of the peptide or restricting the expression of receptors in target cells likely achieves temporal and spatial regulation. For example, defined subsets of ILPs exhibit restricted expression patterns for responding to specific environmental threats such as starvation and heat stress in *C. elegans* (Ritter et al. 2013). In the worm, INS-6 released from ASI neurons functions very differently from INS-6 released from ASE neurons (Chen et al. 2013; Leinwand and Chalasani 2013; Hung et al. 2013; Cornils et al. 2011). These results show that peptide cleavage serves as another important step for regulating insulin signaling (Leinwand and Chalasani 2013). Together, these findings show that tightly regulating the source, target and processing of the peptide signal can achieve specificity in ILP function.

This review highlights the similarities, differences and complexities underlying insulin signaling function in various species. Findings of ILP signaling in *C. elegans* and *D. melanogaster* show that studies in invertebrates can reveal novel functions and mechanisms of insulin action. We propose that these findings provide candidate pathways and new physiological roles for insulin peptides to be tested in the context of the more complex mammalian signaling networks. Moreover, the immense diversity in insulin-regulated neuronal roles observed in invertebrates provides a framework for a similar analysis of insulin or other peptide signals in the mammalian brain. Understanding peptide regulation and function is critical to deciphering the complex mammalian brain (Bargmann 2012). To conclude, we argue that this versatile signaling molecule, insulin, has evolved extensively to take on diverse roles based on context regulating both animal physiology and neuronal functions across species.
